# A Custom Approach for a Flexible, Real-Time and Reliable Software Defined Utility

**DOI:** 10.3390/s18030718

**Published:** 2018-02-28

**Authors:** Agustín Zaballos, Joan Navarro, Ramon Martín De Pozuelo

**Affiliations:** Engineering Department, La Salle—Universitat Ramon Llull, 08022 Barcelona, Spain; jnavarro@salleurl.edu (J.N.); ramonmdp@salleurl.edu (R.M.D.P.)

**Keywords:** smart grids, information and communication technologies, network management, internet of things, security, software-defined architectures

## Abstract

Information and communication technologies (ICTs) have enabled the evolution of traditional electric power distribution networks towards a new paradigm referred to as the smart grid. However, the different elements that compose the ICT plane of a smart grid are usually conceived as isolated systems that typically result in rigid hardware architectures, which are hard to interoperate, manage and adapt to new situations. In the recent years, software-defined systems that take advantage of software and high-speed data network infrastructures have emerged as a promising alternative to classic ad hoc approaches in terms of integration, automation, real-time reconfiguration and resource reusability. The purpose of this paper is to propose the usage of software-defined utilities (SDUs) to address the latent deployment and management limitations of smart grids. More specifically, the implementation of a smart grid’s data storage and management system prototype by means of SDUs is introduced, which exhibits the feasibility of this alternative approach. This system features a hybrid cloud architecture able to meet the data storage requirements of electric utilities and adapt itself to their ever-evolving needs. Conducted experimentations endorse the feasibility of this solution and encourage practitioners to point their efforts in this direction.

## 1. Introduction

Contrary to the fast evolution experienced in the last decade by ICTs, electric power distribution systems have remained exceptionally steady for a long time. Therefore, the advent of the smart grid concept, that is the addition of a telecommunication infrastructure to the electrical domain, has enabled a plethora of new services and opportunities (e.g., accurate grid monitoring, real-time energy consumption and customer-side generation, tracking of overloads, preventive mechanisms for service faults, network balancing to manage the demand, demand response, network self-healing, resilience, etc.), which is actually driving a significant revolution in the energy sector [1,2]. In fact, smart grids are conceived to improve traditional electric power networks in several dimensions [3]—such as information (from scarce one-way data streams to real-time fully meshed big data), business models (from producers and consumers to prosumers), and energy (from fossil-based and centralized production to renewable energies and distributed production)—in order to meet the highest standards of power quality guaranteeing the electric power grid is cost effective and sustainable [4].

Due to the complexity (i.e., stringent levels of service reliability and availability) and magnitude (i.e., large-scale areas) inherent to smart grids, practitioners have recently addressed the digital transformation of power electric networks following a “divide and conquer” approach [4]. That is, by proposing individual and isolated ad hoc solutions for every smart grid matter (e.g., security [5], real time communications [6], internet-scale data management [7]) resulting in a hybrid architecture composed by several software and hardware bricks that, typically, are difficult to put together as a single entity [6] and interoperate [6,8,9] between each other. Moreover, these niche-oriented solutions are built and deployed on dedicated and costly (in terms of both, development time and budget) hardware platforms [5] that present critical difficulties when they need to be updated according to a new smart function or requirement. 

Indeed, the smart grid has brought to the energy domain a new set of actors (e.g., prosumers, aggregators, energy service companies, virtual power plants) that require a continuous upgrading on the features, timings and functionalities offered by the grid to meet market demands, which is a situation never seen before in traditional power electric networks [3]. Therefore, these suboptimal and non-standardized hardware-based building blocks conceived to address individual and thin-scope challenges of the smart grid, are not a good fit any more because of the ever-growing number of smart functions and end-user services that new power electric networks are committed to offer, which is expected to rocket the operating and capital expenditures of maintaining existing solutions [5]. This situation has motivated a latent demand for a standardized and wide-spectrum solution able to entirely meet the mid-term smart grid requirements considering costs, scalability and adaptability to future scenarios.

In this regard, novel software-based architectures recently coined as software-defined networks/anything (SDN/SDxs) [10,11] combined with service composition strategies [12,13] have appeared as a robust alternative to expensive hardware-based systems by offering fine-grained modularity, maintainability and unified management in time-restrictive large-scale systems. In fact, these service oriented architectures (i.e., SDN/SDxs) and service composition [2,6,14] are committed to modularize the features of the system in independent and tiny services (also referred to as SDUs [1]) that can be advertised, deployed, managed, coupled to other software modules, and instantiated on demand remotely. For instance, SDN/SDxs could program a set of highly distributed resources (e.g., smart meters) to be adapted on the fly on their own (also referred to as self-configuration) to meet the requirements of a new service (e.g., new metering policy).

In SDN/SDxs-based architectures, the data plane and the network control plane are natively decoupled [15], which enables the network abstraction, unification, and centralization of its management. This natural decoupling provides grid administrators and system architects with a complete view of the entire architecture, which gives them a safe way to design more scalable modules that are easy to integrate with different middleware upon request, even in real-time (e.g., similarly to what has been proposed in [4,7], a machine-learning based algorithm might be able to observe, learn the normal behavior, react to abnormalities, and preconfigure the grid to operate in a certain pattern). Therefore, SDN/SDxs-based architectures make systems to act like living organisms that adapt themselves to new events increasing their flexibility and easing their management, which would be very convenient features for the smart grid domain.

Overall, the advantages of using software-defined architectures in the context of Smart Grids are found in the following. First and foremost, most of the services and smart functions [2] that are implemented in costly ad hoc platforms will be programmed (and easy to reprogram) on top of a general purpose hardware able to control the sensors of the electric domain and to communicate with other entities of the ICT domain through an heterogeneous high-speed network. Also, these service-oriented architectures can greatly contribute to face the burden associated to the management and configuration of the smart grid—also seen as a complex internet of things (IoT) [16] system—by providing a standard interface to all its modules that natively isolates ICT duties (e.g., data processing in real time, access control) from electric duties (e.g., smart meters, synchrophasors). Additionally, using software-defined systems in the smart grid may enable cybersecurity architects to continuously implement different and adaptive policies on-demand to address the never ending new issues derived from connecting such a critical infrastructure to the internet. Finally, by deploying context-aware and adaptive services results of great interest for distribution system operators (DSOs), since they no longer have to deal with the intrinsic heterogeneity and high dynamicity of the smart grid.

Hence, the benefits of software-defined systems could finally transform smart grids into real (and reliable) autonomous systems able to properly react to any adverse situation in a scalable and software maintainable fashion. The purpose of this work is to propose the usage of SDN/SDxs-based architectures, and hence SDUs, in smart grids to enable an adaptive operation and seamless deployment of electric power infrastructure services. More concretely, it presents the implementation of a SDU-based smart grid’s data storage and management system prototype developed according to the on-field experiences and knowledge collected during the development of the FINESCE (Future INtErnet Smart Utility ServiCEs) and INTEGRIS (INTelligent electrical GRid Sensor communications) FP7 European projects [8,9]. As smart grids generate an overwhelming and valuable amount of information, designing an adaptive, scalable, and modular software-defined system able to store and process all these data becomes crucial.

The contributions of this work are the following:

- A review of the state-of-the-art on the existing software-defined strategies to meet the requirements of smart grids.
- A proposal of a cloud-inspired smart grid’s data storage module designed by means of SDN/SDx and service composition suitable for real-time electric functions.

The remainder of this paper is organized as follows. Section 2 reviews the related work on the evolution of smart grids to software-defined and digital systems from the communications and pervasive computing perspectives. Section 3 introduces a SDU architecture proposal for the smart grid. Section 4 details the customization of the SDU to implement a data management system for the smart grid that inherits the benefits of the hybrid cloud-computing paradigm. Section 5 presents the experimental evaluation of the proposed system. Finally, Section 6 draws the conclusions and future work directions to extend the adoption of software-defined systems in smart grids.

## 2. Related Work

### 2.1. Smart Grid Communication Requirements

The digital transformation of the electrical distribution grid is an increasing need by DSOs and a subject under an intense research study in several fields, ranging from communication and network protocols to cloud computing architectures [17]. As SDN/SDxs-based architectures aim to address the smart grid conception as a whole [18], it is worth reviewing the niche contributions on every domain to put them together as a software-defined ensemble.

As far as the communication and network protocols field is concerned, the smart grid has an inherent heterogeneous nature which forces system architects to consider different telecommunication technologies [19] such as power line communications or radio and wireless networks [6]. Smart grids are deployed in hostile wireless communication environments [20,21], hence, channel status aware protocols [22] (also referred to as cognitive radio techniques [23]) are needed to reduce communication delay [20], meet quality of service (QoS) needs—in terms of delay, bandwidth and data reliability needs, energy harvesting improvement [22] and reliable distributed sensing [24]. Indeed, cognitive radio has emerged as a feasible alternative to reduce power consumption, overcome radio spectrum shortages, and minimize the interoperability issues between heterogeneous communication networks [19].

Unfortunately, these promising technologies are still far from being implemented in the SDN context. Latest attempts on using IEC61850 and SDNs in smart grids have aimed to meet the increasing security risks and threats that the digitalization of the electrical grid encompasses [25]. Indeed, SDNs are envisioned as a potential alternative to tackle the communication difficulties of the smart grid [26]. A prospective view of this idea is presented in [11,26], where an architecture in which the SDN controller links the communications between the management interface (i.e., the supervisory control and data acquisition) and the end devices (i.e., intelligent electronic devices and remote terminal units) is proposed by means of different use cases (e.g., dynamic virtual machines layer to control grid applications). However, despite these latest advances, it is not yet possible to transparently and proactively operate a heterogeneous energy network composed of a smart grid that integrates different renewable generation sources and consumer models in an efficient and effective way. In this regard, the possibilities of the SDN application to the smart grid development and some concerns about cybersecurity are highlighted [26].

Indeed, one of the major architectural changes of smart grids is the inclusion of renewable energy systems, also referred to as distributed energy resources (DERs), as an active part of the electric network. Hence, classic centralized schemes used to manage (i.e., protect, control, and operate) generation resources in traditional electric networks are not suitable for smart grids due to their inherent distributed nature. Therefore, novel communication standards (e.g., IEC 61850), together with the new communication features brought by ethernet have emerged as a promising alternative to evolve the management and protection policies in modern electric networks. Also, the inclusion of the DER paradigm in the smart grid has some implications on the protection of the electric network at the distribution layer. Specifically, advanced relaying techniques used to protect High Voltage networks in traditional electric grids need to be used in distribution networks as well in the context of the smart grid. As shown in Table 1, these relaying schemes require exchanging messages between different points of the grid with stringent reliability (e.g., 99.999% in services related to DER and demand response [27]) and latency specifications (e.g., 20 ms–15 s in services related to DER and demand response [27]), which forces the communications layer to meet these requirements too in case of failure.

The extension of the concept of smart grids to distribution networks faces the problem related to the difficulty of designing an ICT infrastructure that covers the whole power distribution network with the required high reliability and low delay.

Hence, the aforementioned protection relays that have been used in old high voltage electric networks and their functionalities are now needed at the distribution level of the smart grid, have evolved to what has been coined as intelligent electronic devices (IEDs). These systems are designed to control relays, record faults, report faults, measure the grid, and protect the network in a fast and efficient way. Such efficiency is achieved by the distributed nature of DER and its associated IEDs, which is a great improvement over the traditional centralized schemes found in classic electric networks.

Indeed, the protection, control, and operation of the DERs can be best seen and addressed as a set of software services—thus enabling practitioners to improve the automation of the smart grid management while diminishing the operational costs—that need to be deployed in a large-scale and heterogeneous distributed system (i.e., the smart grid) [11]. As a first step toward this idea, authors have analyzed different communication strategies to deploy, operate, and manage services in a smart grid by means of what has been coined as software-defined utility (SDU) [11]. One of the greatest challenges in this software-defined approach is to keep the aforesaid stringent reliability, delay, and latency demands [5,11] inherent to the electric domain (see Table 1). For instance, as discussed in [5], there are services such as those related to active protection, analysis & monitoring, or regulations and command, that are difficult to successfully implement in real world distribution networks by means of ethernet or even IEC 61850 services. However, an example of adaptive protection was proposed in [28], in which the authors experimented with GOOSE for the communication between IEDs, combining it with several protection schemes. This and other smart grid functions can be deployed over the system in a gradual way, integrating existing hardware or increasingly providing them by software modules that control sensed parameters (i.e., voltage, current) and actuate when specified thresholds are surpassed.

### 2.2. Smart Grid in Clouds

The adoption of the cloud-computing paradigm in organizations and enterprises has become a reality during the last decade. Certainly, the advent of hybrid clouds that combines the benefits of public and private clouds has contributed to the consolidation of this approach [29]. This new resource exploitation model enables companies to elastically adapt their internal infrastructure (i.e., private cloud) to the incoming workloads with third party resources (i.e., public cloud). Additionally, this also enables system architects to appropriately balance incoming workloads in terms of privacy or efficiency. For example, the private side of the hybrid cloud be used to conduct processing operations with critical data and the public side could be used for non-sensitive data transactions. However, the complexity associated to the hybrid cloud approach prevents practitioners from fully exploiting it [30]. This complexity is mostly derived from the interconnection and integration of two (or more) isolated infrastructures independently managed, which typically requires a superior entity (also referred to as resource orchestrator) with a global view (e.g., dashboard [31]) of the whole system. This decision-maker is in charge to automatically decide when and whether to use the private or public side of the infrastructure.

As far as cloud computing architectures for the smart grid are concerned, it has been shown [32] that the features (i.e., scalability, on-demand storage and computing resources, elastic services) offered by the cloud paradigm are especially appealing for the smart grid context—as long as their associated cybersecurity threats are properly addressed. The pros and cons on using each cloud computing deployment model (i.e., public cloud, private cloud and hybrid cloud) as well as their exploitation models (i.e., software as a service, platform as a service, and infrastructure as a service) for the smart grid domain are thoroughly discussed in [32] from the technical and security perspectives. It turns out that the hybrid cloud approach is the most suitable for the smart grid since it enjoys the benefits of both private (i.e., security) and public clouds (i.e., availability and operational costs) [33]. In [4], a custom cloud-inspired distributed storage system is presented to address the data storage demands of smart functions. This proposal takes advantage of the cloud philosophy to adapt its architecture [7] to the smart functions demands in terms of throughput, data volume, reliability, and security [34]. To further boost the interoperability and seamless integration of the different components of a cloud-computing infrastructure, the idea of a software-defined cloud is explored in [35]. Specifically, authors propose a generic architecture that consists of splitting the cloud into seven virtualization-based layers (i.e., physical layer, compute virtualization, network virtualization, security virtualization, virtual pools, control layer, and application layer) and successfully assess their proposal using the Mininet simulator, which is still a bit far from the reality faced by the smart grids.

Following the idea of decomposing large-scale systems into small software components easy to interoperate, in [18] authors propose to address the software-defined smart grid as the composition of four modules: a SDN to address communications, a software-defined storage (SDStore) to address data-related concerns, a software-defined security (SDSec) to address cybersecurity matters, and a software-defined internet of things (SDIoT) to address the management of devices [16]. It definitely represents a valuable contribution on this topic that exposes a different way and model to analyze the smart grid infrastructure. However, the presented architecture is generic and abstract, and it still needs to define more concretely how most of the exposed subsystems (i.e., SDStore, SDSec, SDIoT) can be deployed and managed.

Overall, after reviewing all of these architecture proposals, there is an increasing need on building and deploying a software-defined system able to incorporate all the aforesaid individual advances (i.e., context-aware real-time communications, cybersecurity, cloud storage) in a real-world smart grid. The following section details the architecture of the software-defined utility used in the FINESCE European project [8], which is an alternative approach to the software-defined smart grid inspired by the module decoupling proposed in [18,35], and implementing a hybrid cloud data management system (HCDM) as envisaged by [33,35].

One of the objectives in this paper is to propose a hybrid approach to handle real time data collected from the smart grid domain at the distribution level (e.g., metering, charging stations of electric vehicles, etc.). This system will store data from historic logs at the private side of the hybrid cloud and use the public source to outsource bursts and answer low-priority queries. This proposal will be tested in a real-world environment composed by data centers placed in both public and private clouds. As it will be shown, data will be replicated to different sites (belonging to public and private clouds) to boost the system availability, minimize the overall latency, and meet the cyber-security restrictions posed by smart grid experts.

The complexity of this proposal relies on the fact that distributing smart grid services among a distributed architecture composed by a public and a private cloud, keeping the specified quality of service (QoS) [36] is far from trivial. This challenge is addressed by automatically inferring a set of rules that define the boundaries of time and cost of service allocation, prioritization policies, and data placement. These rules are used to decide which is the most convenient node to run a service in a given situation [29].

## 3. Software Defined Utility Architecture

**Figure 1:**
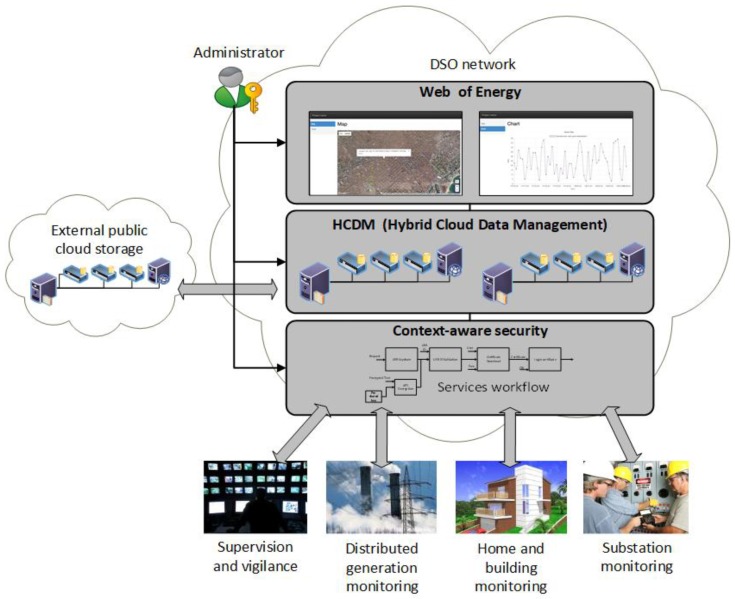
Main software-defined utility modules: context-aware security, Hybrid Cloud Data Management (HCDM) system, web of energy.

The aim of the SDU is to enable the digital transformation of the smart grid towards an intelligent and programmable grid. As with other software-defined architectures [18,35], our proposal of SDU for the smart grid also inherits the concepts, philosophy, and strategies born in the software-defined systems and service composition fields. Therefore, our proposed SDU architecture is composed of three main independent modules (see Figure 1). Each module is detailed in what follows.

- Context-aware security. This module is aimed to individually provide the needed security level for the proper operation of every smart function. Cybersecurity policies are served on demand by means of service composition and continuously revised by the module itself. For instance, for the use case of smart metering, this module can update all the encryption keys of the smart grid once an unauthorized access to the metering infrastructure has been detected.
- Hybrid cloud data management: This module provides a data storage and processing system that intrinsically adapts to the smart grid’s topology in a scalable and flexible way. Specifically, it implements a software-defined version of [4,37], resulting in a distributed (and distributable) storage architecture that uses epidemic data propagation to replicate collected data in public and private clouds (i.e., hybrid cloud [33]). In addition, it implements an algorithm (also referred to as orchestrator) to decide whether collected data should be stored at the private cloud or could be placed at the public cloud [7]. Such decisions are made considering the smart functions requirements (e.g., reliability, delay, cybersecurity) associated to the collected data [27].
- Web of energy: This module provides a ubiquitous (i.e., web-based) monitoring interface [38] that enables a seamless management of the whole IoT [16] architecture of the smart grid. In addition to provide a mechanism to communicate humans and machines, it also enables the interactions between those IoT resource-constrained and small devices (i.e., machine to machine) through the HTTP protocol. Note that the aforementioned context-aware security module might decide to add an extra layer of security by switching from HTTP to HTTPS given the device features, network status, and smart function under execution demands. This is done through an open API that both couples and decouples all the modules, as typically done in SDN/SDXs systems. Hence, this module also acts as a bridge between the distributed storage layer—that takes care of all the smart grid’s big data concerns—and the context-aware security layer—that gives the necessary access control and cybersecurity mechanisms.

However, despite the benefits associated to the service composition paradigm, implementing these three logical modules in existing intelligent electronic devices (IEDs) that currently populate the smart grid becomes unfeasible. Nowadays, deployed IEDs that mostly implement the ICT side of the smart grid are closed devices that, once approved for operation, certified, and installed are difficult to upgrade with new advancements (e.g., novel cybersecurity policies or data replication algorithms) as experimental devices do [4]. Hence, for the sake of the FINESCE European project [8] we have designed the FIDEV (FInesce DEVice): a novel software-defined device [1] that includes the three aforementioned modules and is aimed to be the natural replacement of existing IEDs. Hence, FIDEVs behave as a frontier between the electric domain and the ICT layer. Note, that in addition to the three modules that conform the SDU core, each FIDEV implements a communications subsystem (e.g., cognitive radio [19]) that enables the heterogeneous network coexistence. It is worth noting that opposite to other software-defined architectures for smart grids [18], this proposal (1) hard codes the SDN layer into the device implementing the SDU (i.e., FIDEV) and (2) combines the SDN and IoT controllers into a single layer (i.e., web of energy). In the context of the FINESCE European project, these decisions have resulted in a faster development time of the software components, ease of deployment and integration, and greater stability.

Overall, a FIDEV can be best seen as a single, yet distributable and interconnected element that belongs to a fog computing architecture that, thanks to the three aforementioned modules, is able to solve existing and future real time smart functions [1] by offering the following features: cyber secure access control, privacy management, data storage, reliable and high-speed communication, remote terminal units (RTU), electric data collectors. In this regard, [29] has presented a cloud parameterization according to a set of indicators (e.g., response time), that models its behavior. As measuring these indicators might be tricky and poorly generalizable, authors have conducted a qualitative assessment of the presented metrics.

## 4. The SDU Data Management System

This section presents the implementation of a distributed data storage system aimed to store, process, and serve the required data for smart functions in the smart grid by means of the proposed FIDEVs and their SDU paradigm.

The main goals of the proposed data management subsystem are: (1) ensure high-availability and reliability on stored data, (2) minimize the delay between user requests and data delivery at any point (e.g., utility facilities, end-users), (3) provide the smart grid with a cyber-secure strategy to share its data with external players and (4) enable service applications and data to be flexibly and smoothly moved over the smart grid to take advantage of the available resources in both public and private clouds. In fact, promoting (or replicating) information and data from local sites to the public cloud will, additionally, contribute to increase their availability and reliability, reduce the response time (i.e., data will be stored close where it will be needed), and help to change the data privacy degree (i.e., removing data from the public cloud when they are too sensitive), including local resources running out of capacity (i.e., provisioning more resources from the cloud). Hence, to enable the gradual incorporation of cloud resources to the Distribution System Operator (DSO) infrastructure an epidemic data replication protocol inspired by the fog computing paradigm has been implemented.

**Figure 2:**
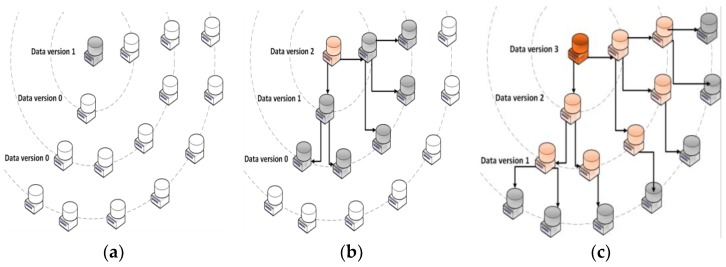
Epidemic replication protocol with three layers. Nodes from public or private clouds can be added to any layer at will. (**a**) Version 1 of data is generated at the core layer. (**b**) Version 1 is propagated to the next layer and a new version (i.e., version 2) is generated at core layer. (**c**) Data from Version 1 and 2 are propagated to their subsequent layers and a new version (i.e., version 3) is generated at the core layer.

This epidemic replication protocol (see Figure 2) [37] is inspired by the one used in the INTEGRIS European project [4]. Specifically to avoid flooding, every time a new datum is generated, each node of the whole network (i.e., FIDEV) periodically propagates its data to its defined neighboring nodes (also referred to as replication layers), which drives to the eventual consistency model typically found in cloud-computing architectures. Hence, nodes belonging to the replication layer where a datum was generated will own staler data than nodes belonging to closer replication layers. This strategy allows to balance low-delay data requests (i.e., give a faster response) to the core layers and, thus, keep QoS agreements. Each layer can be composed by both: nodes (i.e., FIDEVs) spread over the electrical distribution network (i.e., private cloud) or general purpose nodes hosted in public clouds. That is, this epidemic replication chain [37] can be composed by nodes from both public and private infrastructures that can be added and removed at will in real time, which translates into a hybrid cloud-computing approach. In this way, data will be replicated in several geographically distant locations, which will make it available from anywhere with a reasonable latency. The selection of which nodes belong to each replication layer must be taken according to the services that will be deployed over them (e.g., real-time consumption monitoring, recovery of lost information, network self-healing).

Hence, smartly defining the replication layers the smart grid manager can select the most appropriate place to store every datum: either locally at the facilities owned by the utility company or by means of alien public cloud providers (in the latter case, privacy policies will be enforced). Indeed, coming up with the optimal configuration of resources topology in a hybrid cloud storage infrastructure may result in great savings [39]. However, it is difficult [40] to select the most convenient infrastructure to place a given datum in those scenarios where several geographically interleaved clouds are involved, due to the large number of parameters to be considered (e.g., economic cost, data transfer time, throughput) and the lack of a reliable model to know the cloud behavior in advance [29].

Therefore, the current version of the HCDM includes an orchestrator that implements a set of rules that cover basic parameters such as privacy, throughput, distance to client, and smart function data requirements to move data between clouds. Considering the large number of situations and possible combinations for these HCDM status parameters, the data migration and placement rules are built as follows.

First, the system is exposed to different real-world situations for long periods of time (i.e., days). These example scenarios are proposed by industry experts and are aimed to model distinct and representative events (e.g., a cloud runs out of resources, a cloud becomes insecure, all clouds are over provisioned, etc.). Meanwhile, system parameters and statistics are accurately measured and stored every 10 s. Next, a decision tree is built from all collected data using a standard C5.0 classifier. Next, a set of human-readable production rules is derived from the decision tree as done in [41]. Finally, this set of rules is validated by domain experts who fine-tune every rule. However, more sophisticated solutions [42] should be considered in the near future to address situations with more clouds and highly dynamic scenarios where online learning might be needed.

**Figure 3:**
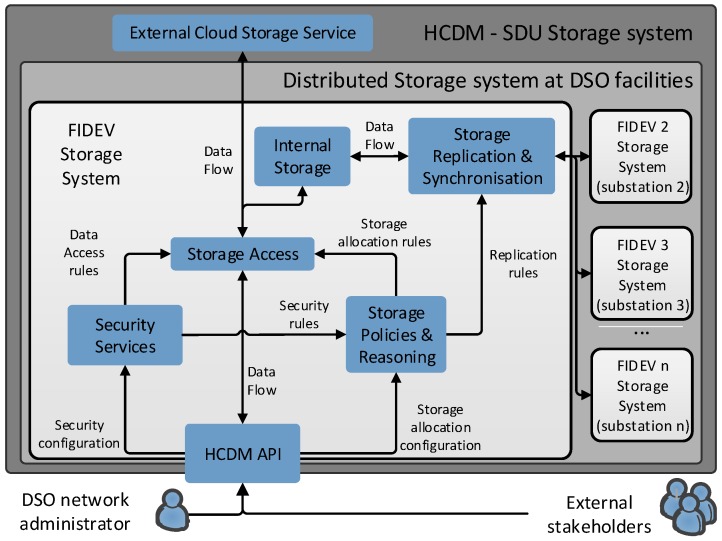
Service composition diagram of the HCDM.

As shown in Figure 3, the whole HCDM module has been implemented following the service composition paradigm. An open RESTful “HCDM API” service has been implemented to enable external stakeholders and DSO network administrators access the FIDEV device. This API defines how to interact with the SDU storage system and abstracts the system to the smart grid functions that could be deployed above it. Therefore, the proposed architecture does not link the user to a specific application over it. A “security services” module borrowed from the context-aware security subsystem (see Figure 1) provides access control and implements data privacy policies (e.g., encryption). A “storage reasoning & policies” service configures the topology of the epidemic replication (e.g., promoting nodes along the replication chain, excluding nodes). A “storage access” service orchestrates where data will be stored storage by deciding whether to place them in the public or private infrastructures. An “internal storage” service is used as a metadata manager and fast access cache. A “storage synchronization & replication” service is used to physically store data and keep their consistency. Hence, this implementation successfully combines services from the three different layers in a seamless and flexible way. Following this strategy, modifying, removing, or adding new services to build other smart grid modules and upgrade existing ones would be more than feasible. However, it has to be said that the HCDM module also represents a single point of interaction of the used with the data generated, but, as represented in Figure 3, it could be combined with any other external storage system, cloud-based or even novel blockchain-based distributed storage systems such as IOTA [43].

## 5. Experimental Evaluation

**Figure 4:**
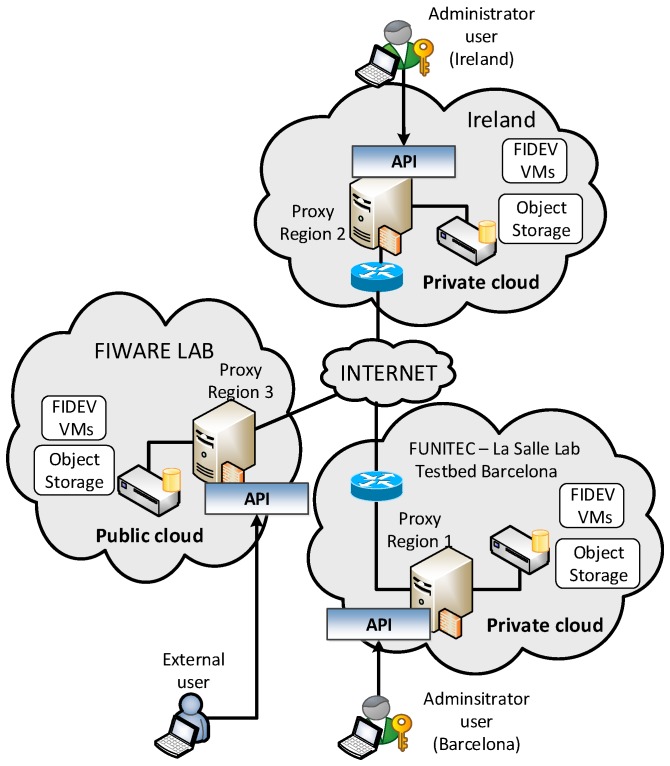
Test environment built on the FINESCE project facilities. There are three physical locations with three FIDEVs (FInesce DEVices) hosted at each location. The proposed HCDM Application Programming Interface (API) under evaluation is running at all FIDEVs. Each FIDEV is in charge of ten smart meters.

After detailing the internals of the HCDM module, a real-world trial scenario has been set up to experimentally evaluate the benefits of this proposal. In this regard, we have used the facilities provided by the FINESCE European project (Figure 4) that consists of two laboratories placed in Barcelona and Ireland plus a public cloud (FIWARE Lab in Figure 4).

The aim of this scenario is to share electric data between the clients attached to both locations and publish them to clients connected to the public cloud. Hence, we have deployed the aforementioned HCDM system to maintain data generated by ESB (the Electricity Supply Board is a public electricity company from the Republic of Ireland) replicated both locally and in the hybrid cloud (i.e., either at the FIWARE Lab or at Barcelona).

The proposed experiment aims to test whether the proposed system can keep the QoS (delay and latency) for the smart grid data under three different situations. In this regard, three different replication regions have been defined: Ireland, Barcelona, and Public Cloud. We have considered that the QoS will be maintained as long as the overall performance of all nodes is under 100%. The proposed experiment aims to evaluate the overall data access latency under different situations.

On the one hand, four different load patterns have been defined in order to evaluate time restrictions:

- System idle (5% data read operations, 5% data update operations and 90% “do-nothing” operations).
- Medium load (50% data read operations and 50% data update operations)
- Update intensive load (20% data read operations and 80% data update operations) and
- Read intensive load (80% data read operations and 20% data update operations).

On the other hand, the data encryption service from the Context-aware security subsystem has been remotely installed and uninstalled—by means of an OSGI service framework—several times during the trial to assess its effects on the data access latency. When this security service is enabled, all data written/read to disk is encrypted/decrypted using AES and a 256-bit key. Additionally, the HCDM module has been configured with three different replication regions have been defined: Ireland, Barcelona, and Public Cloud.

**Figure 5:**
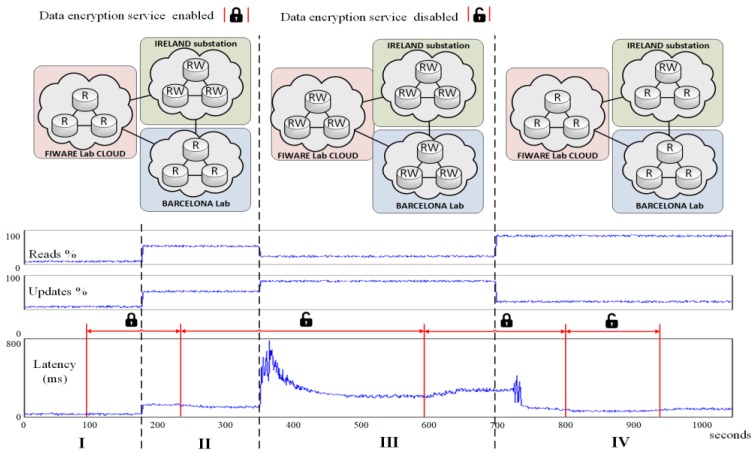
Evolution of the FIDEVs when exposed to different situations. Ratio of data requests (reads %), data generation (updates %), and overall latency from all the nodes are depicted on the bottom. FIDEVs configurations are depicted on top.

In the first scenario (I in Figure 5), it has been defined that only Ireland is generating new data from the smart meters directly attached to the FIDEVs. Thus, the newest data from Ireland should be stored in the private part of the hybrid cloud while the older data should be replicated at the other layers (i.e., private cloud at Barcelona and public cloud at FIWARE Lab). Note that when a FIDEV generates data, it is labeled as “RW” in Figure 5 and when it only receives data requests, it is labeled as “R”. Hence, the public cloud side is initially committed to (1) tackle queries that can deal with stale data (or can afford some time to receive data from the core layer) and (2) provide on-demand extra storage resources [29] in those situations where the private cloud is unable to handle more load (also referred to as burst outsourcing model) [44]. This scenario has been configured with such a low load pattern to obtain a best-case value for the latency parameter. In this situation, we have measured that the overall latency among the read/write operations issued by all the nodes is around 33 ms. At second 100, the encryption service has been enabled. It can be seen that it has a negligible impact on the latency due to the low amount of operations running at each node.

In the second scenario (II in Figure 5), we have emulated a situation in which smart functions are generating the same amount of read and write operations. In this case, the latency rises up to 114 ms. Later, at second 223, the data encryption service is uninstalled, which reduces the latency to 90 ms. We have measured that it takes 22 s from the moment that the uninstall command is issued until latency is stabilized (less than 10% variation).

In the third scenario (III in Figure 5), we have emulated a situation in which several smart functions suddenly start running at a time and thus, all the FIDEVs start generating large amounts of data. Hence, the epidemic data replication system must reconfigure itself to meet the new update intensive load [37]. It can be seen (second 354) that the previous configuration rockets the latency, which triggers the replication scheme reconfiguration event. We have measured that it takes 151 s from the moment that the reconfiguration event is triggered until latency is stabilized to 203 ms (i.e., the new replication scheme becomes full operative). Later, at second 600, the data encryption service is installed again, which increases the latency up to 241 ms. We have measured that it takes 61 s from the moment that the install command is issued until latency is stabilized. Such increase on the installation time is due to the high congestion of the network.

Finally, in the fourth scenario (IV in Figure 5), we have emulated a situation where most of the smart functions suddenly stop running and a large number of external stakeholders are retrieving information from the stored data (second 700). Therefore, the amount of generated data (due to the smart functions) decreases and the number of data requests increases (e.g., there are several smart functions running at a time). After all the queued update intensive operations are executed (second 727) the latency dramatically decreases, which triggers another replication scheme reconfiguration event. This reconfiguration process causes some glitches on the latency, but once the process is done (it takes 28 s) the latency stabilizes to 83 ms. Later at second 223, the data encryption service is uninstalled again, which reduces the latency to 48 ms. We have measured that it takes 16 s from the moment that the uninstall command is issued until latency is stabilized. Finally, at second 940, the data encryption service is installed again, which increases the latency up to 78 ms. We have measured that it takes 18 s from the moment that the install command is issued until latency is stabilized.

Although the physical architecture of the FIDEVs remains stable (i.e., no communication fading has been introduced), the system has logically adapted its epidemic replication chain according to the incoming load [37]. Specifically, in situation I and II, the replication chain was Ireland–Barcelona–FIWARE Lab. In situation III, the system has needed to merge the three regions into a single one in order to meet the peak load. In situation IV, the system has defined two regions: one composed by the single FIDEV at Ireland generating data and the other region composed by the remaining nodes (i.e., primary copy replication [37]).

To sum up, the latency ranges from 33 ms to 241 ms once the system is stabilized depending on the load pattern, the replication scheme, and security policy status. The cyber security service installation and uninstallation time ranges from 18 to 61 s and has a strong dependency on the network congestion and load at each node. Finally, the HCDM replication reconfiguration scheme time ranges from 28 s to 151 s. Recall that moving from “RW” to “R” is cheaper than moving from “R” to “RW” due to the replica synchronization overhead. However, these few minutes are tolerable when compared to the cost of manually updating the IEDs of a whole country (which would take few days). Actually, these numbers are in the same order of magnitude of other related presenting the application of SDN to control smart grid communications [25] and exhibit reconfiguration times of thousands of milliseconds. In preliminary communication tests (i.e., no load was generated) from the system to different IEDs, the maximum contact time was 4 ms in local scenarios. However, if there is a long distance, the time will increase considerably over 20 ms and the IEDs latching signals latency will be reduced about 70%, in comparison with traditional wiring [44].

Overall, this experiment exhibits the advantages of the proposed epidemic data replication protocol and shows the ability of the system on adapting to incoming loads. The development and deployment times of this software-defined architecture have been considerably smaller (i.e., several orders of magnitude) than the ones obtained in similar projects [1,37].

## 6. Conclusions and Future Work

This work presents the evolution and redesign of the distributed storage architecture to address the challenges arisen at the distribution level of modern smart grids. An initial version of the proposed epidemic replication protocol was successfully deployed in the scope of INTEGRIS, but its complexity, high number of dependencies, and long development times have driven practitioners to evolve to what has been presented in this paper by means of service composition and software-defined architectures in the context of the FINESCE project. In addition to the operational advantages of using the service composition paradigm, this new version of the data management system addresses the access control to stored resources, natively manages passwords and encryption keys, and is fully integrated to a real cloud computing infrastructure.

The proposed HCDM system is built inside a SDU that is composed by three artifacts: concept (web of energy, HCDM, and context-aware Security). For the implementation of the data management system, services borrowed from the Web of Energy enable users to access the data storage system, services borrowed from the context-aware security guarantee that cybersecurity policies are being respected, and services borrowed from the HCDM ensure that data are properly stored. This module decoupling is inspired by existing proposals on using software-defined architectures for the smart grid. To the best of our knowledge, this is the first real-world implementation of an operating software-defined system that addresses the data requirements of the smart grid by taking advantage of the hybrid cloud-computing paradigm.

Overall, the proposed SDU-based data management module addresses the smart grid data requirements by (1) providing high-availability and reliability on data through an epidemic replication protocol, (2) minimizing latency by proactively replicating data where it will be needed, (3) injecting cyber security services to provide different degrees of privacy and access and (4) moving services and applications on demand. Moreover, to speed up the transference of new services and applications (e.g., large virtual machines), smart grid administrators can take advantage of the epidemic nature of the proposed HCDM system and split the service to be transferred into small pieces and let the replication protocol to populate all the system in parallel. Indeed, computing intensive smart functions might also take advantage of this replication strategy and natively conduct computations following the map/reduce approach (i.e., initially computing isolated parts of the function and then propagating and aggregating the results over the network).

Nonetheless we have found that the proposed hybrid cloud-based approach requires considerable effort to obtain the optimal data replication topology—specially in large scale systems—due to the diverse data contexts and cybersecurity constraints associated to the smart functions. An inappropriate replication configuration usually derives in an overusage of a set of FIDEVs or leaving some FIDEVs underused. Therefore, defining and deploying a system able to predict the system status and automatically define a set of configuration rules is planned as the future directions of the authors.

Furthermore, future work directions shall include a revision of the standardization and performance analysis of existing protocols to communicate services in a software-defined system, implementation of other smart grid modules by means of service composition, and an enhancement of the primitive data orchestrator proposed in this work.

## Figures and Tables

**Table 1:** Real-time electrical functions requirements.

Function Class	Value Signal	Transfer Time	Reliability Level
Active protection functions	Block & trip signal	≤20 ms	Very High (99.999%)
Command and regulations	O/C command load shedding Peak shaving	≤2 s	High (99.99%)
Monitoring and analysis	Analogical & digital TVPP	≤2 s	High (99.99%)
Advanced meter & supply management functions	Energy meas. supply management commands, alarms	≤5 m ≤10 s	Low (99%)
End-to-end info. exchange and management	Energy meas. Other signals	≤5 m ≤5 s	Medium (99.9%)
